# Statistical method to compare massive parallel sequencing pipelines

**DOI:** 10.1186/s12859-017-1552-9

**Published:** 2017-03-01

**Authors:** MH. Elsensohn, N. Leblay, S. Dimassi, A. Campan-Fournier, A. Labalme, F. Roucher-Boulez, D. Sanlaville, G. Lesca, C. Bardel, P. Roy

**Affiliations:** 1 Service de Biostatistique-Bioinformatique, Hospices Civils de Lyon, 162 avenue Lacassagne, F-69003 Lyon, France; 2 Université de Lyon, Lyon, France; 3 Université Lyon 1, Villeurbanne, France; 4 CNRS UMR5558, Laboratoire de Biométrie et Biologie Evolutive, Equipe Biostatistique Santé, Villeurbanne, France; 5 Service de Génétique, Hospices Civils de Lyon, Lyon, France; 6 Centre de Recherche en Neurosciences de Lyon, CNRS UMR 5292, INSERM U1028, Lyon, France

**Keywords:** Statistical methods, Massive parallel sequencing, Next-generation sequencing, Pipeline comparison, Sensitivity, Specificity

## Abstract

**Background:**

Today, sequencing is frequently carried out by Massive Parallel Sequencing (MPS) that cuts drastically sequencing time and expenses. Nevertheless, Sanger sequencing remains the main validation method to confirm the presence of variants. The analysis of MPS data involves the development of several bioinformatic tools, academic or commercial. We present here a statistical method to compare MPS pipelines and test it in a comparison between an academic (BWA-GATK) and a commercial pipeline (TMAP-NextGENe®), with and without reference to a gold standard (here, Sanger sequencing), on a panel of 41 genes in 43 epileptic patients. This method used the number of variants to fit log-linear models for pairwise agreements between pipelines. To assess the heterogeneity of the margins and the odds ratios of agreement, four log-linear models were used: a full model, a homogeneous-margin model, a model with single odds ratio for all patients, and a model with single intercept. Then a log-linear mixed model was fitted considering the biological variability as a random effect.

**Results:**

Among the 390,339 base-pairs sequenced, TMAP-NextGENe® and BWA-GATK found, on average, 2253.49 and 1857.14 variants (single nucleotide variants and indels), respectively. Against the gold standard, the pipelines had similar sensitivities (63.47% vs. 63.42%) and close but significantly different specificities (99.57% vs. 99.65%; *p* < 0.001). Same-trend results were obtained when only single nucleotide variants were considered (99.98% specificity and 76.81% sensitivity for both pipelines).

**Conclusions:**

The method allows thus pipeline comparison and selection. It is generalizable to all types of MPS data and all pipelines.

**Electronic supplementary material:**

The online version of this article (doi:10.1186/s12859-017-1552-9) contains supplementary material, which is available to authorized users.

## Background

Today, various sequencing methods are available for routine sequencing. The first method was that of Sanger [1]; it was used in many “historically significant” large-scale sequencing projects. Until recently, for diagnostic purposes, only a few number of genes could be sequenced by Sanger method. Actually, this method is costly, time-consuming, and less practical than more recent methods for sequencing all genes potentially associated with a given disease.

By 2000, less expensive and more automated sequencers were designed: Massive Parallel Sequencing (MPS) --also called Next Generation Sequencing (NGS)-- came to reality [2, 3]. MPS platforms decreased drastically the time and costs associated with comprehensive genome analyses. These platforms allow sequencing specific genomic regions or whole genomes to investigate associations between diseases and genomic variants (single nucleotide variants –SNVs–, insertions, deletions, or balanced and unbalanced structural variations). The possibility of sequencing a high number of genes or a whole genome for a limited cost led to the use of MPS technology for screening mutations in routine diagnosis or research [4].

Different MPS technologies based on different DNA properties are now available (Illumina, Ion Torrent, Roche, etc.). These technologies were compared by several authors [3, 5–7]. In the present study, we focused on Ion Torrent PGM^™^ (Life Technologies, CA, USA; now became Thermo Fisher Scientific, Waltham, MA), a semi-conductor sequencer that detects the proton(s) released when nucleotides are incorporated during DNA synthesis. This sequencer does not require fluorescence or scan camera; it is thus faster, smaller, and less expensive than others, such as Illumina MiSeq (Illumina^R^, San Diego, CA, USA) or 454 GS Roche Junior (Roche Applied Science, Indianapolis, IN, USA).

The advent of MPS entailed the development of a great number of bioinformatic tools to analyse the high-dimensional data generated [8]. Academic and commercial tools have been proposed, the latter being often academic software programs with pleasant interfaces and parameters adapted to specific sequencing technologies. These bioinformatic tools, called pipelines, determine the positions of mutations in a patient’s sequence upon comparison with a reference sequence. The two main steps in the majority of pipelines are: read alignment on a reference sequence (e.g., Bowtie [9], MAQ, [10], BWA [11], or SOAP [12]) and variant calling (e.g., GATK [13], SAMtools [14], or FreeBayes [15]). Any pipeline may be used to analyse MPS data; however, choosing between pipelines is very difficult and requires objective comparisons.

Several recent papers compared the results of various pipelines and most considered Sanger sequencing as the gold standard and reference for NGS pipeline validation [16–21]. Nevertheless, because Sanger sequencing is not a “perfect” gold standard, several studies have used instead simulated or artificial data [22, 23]. All these studies determined the number of false positives (FPs) and calculated sensitivity. To our knowledge, no statistical modelling was yet specifically developed to compare pipelines.

The aim of the present study was the development of a statistical method to evaluate the quality of the results given by various MPS pipelines. In a first part, this statistical method compares two pipelines without using a gold standard. In a second part, two pipelines are compared with Sanger sequencing as gold standard.

## Methods

### Source of data

The analysis concerned a panel of 41 genes involved in epilepsy and 43 epileptic patients. Among these, 30 patients were also sequenced by the Sanger technique for 1 to 3 genes selected according to the clinical symptoms.

All sequencing reactions were carried out in a single laboratory (Department of Genetics, Hospices Civils de Lyon, France).

### Gene sequencing

The molecular genetic analyses were performed after obtaining informed consent from the patients or legal guardians. DNA was extracted from EDTA-preserved whole blood using Nucleon BACC3 kit (GE healthcare Life Sciences, Buckinghamshire, UK).

#### Massive parallel sequencing

The library for each patient was prepared with a Haloplex® custom kit (Agilent Technologies, Inc, Santa Clara, CA) according to the manufacturer’s instructions. Probes were designed to target 41 candidate genes involved in epileptic disorders. The sequencing was carried out using an Ion 318™ Chip on the Ion Torrent PGM™ (Life Technologies) and the PGM™ Sequencing 200 Kit. Enriched template-positive Ion PGM™ spheres were prepared by emulsion PCR with the Ion OneTouch™ 2 System (Life Technologies). One unmapped bam file per patient was obtained; it contained all non-aligned patient fragment sequences (reads). These unmapped bam files were transformed into Fastq files with the plugin fastqcreator.

#### Sanger sequencing

Sanger sequencing was carried out by conventional dideoxy sequencing with amplification of exons and exon/intron junctions followed by direct sequencing using Big-Dye Terminators (Life Technologies). Sequences were loaded on an ABI3730XL sequencer and analysed with SeqScape software, v2.5.

### Bioinformatic analysis

Two pipelines were used: an academic pipeline (BWA-GATK) and a commercial pipeline (TMAP-NextGENe®).

The BWA-GATK pipeline was designed according to recommendations from Broad Institute [24] and using default parameters. The fastq files used at the beginning were constructed from unmapped BAM files given by Ion Torrent Suite. Briefly, its main steps are: (i) alignment of reads to the reference genome (Human genetic sequence reference, Hg19) using BWA-MEM algorithm, v0.7.6a; (ii) realignment around indels using GATK; and (iii) variant calling using GATK HaplotypeCaller. Variants with at least 10× sequencing depth and located within the sequenced region (defined in the bed file) were retained in the final VCF file. No other filter was applied.

The TMAP-NextGENe® pipeline includes two main steps. First, the reads are aligned to the same reference genome (Hg19) using TMAP (Torrent Mapping Alignment Program, the aligner provided by Life Technologies in the Torrent Suite). The TMAP includes several algorithms: BWA-short [11], BWA-long [25], SSAHA [26], and Super-maximal Exact Matching [27]. It uses a two-step approach: reads that do not align during the first step are passed to the second step with a new set of algorithms and/or parameters. Then, alignment files (bam files) are loaded into NextGENe® to carry out variant calling. Default parameters were used with both programs (TMAP and NextGENe®). Variants with at least 10× sequencing depth and located within the sequenced region were retained in the final VCF file.

#### MPS vs. Sanger

For a relevant comparison, when Sanger sequencing was used, in each patient, only bases located in regions sequenced by both MPS and Sanger sequencing were considered in the analysis.

### Statistical analysis

#### Contingency table definition

Each chromosomal position on the reference genome (Hg19) was considered as the statistical unit.

**Fig. 1:**
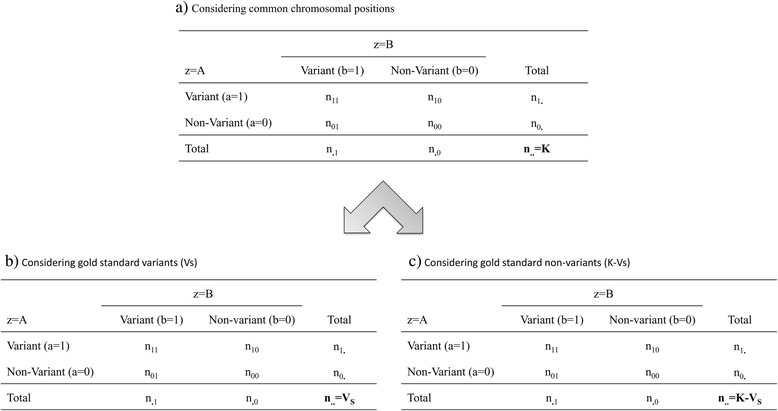
Four-cell contingency tables for pipeline agreement on chromosomal positions. Panel **a** Pipeline comparison (A vs. B) without gold standard. Panel **b** Pipeline comparison of gold standard variants. Panel **c** Pipeline comparison of gold standard non-variants. K: number of chromosomal position considered. *Vs*: number of gold standard variants

For a given patient, a given pipeline z∈{A, B}, and a given chromosomal position k = 1,…,K, let *X*_*zk*_ be a random variable taking value 1 when a variant is detected at position k and 0 otherwise. A 2 by 2 table for agreement on variant identification can then be built using the following Eq. (1) (Fig. 1a):

1 $$ {n}_{ab}={\displaystyle \sum_{k=1}^K\left( I\left({X}_{Ak}= a\right)\times I\left({X}_{Bk}= b\right)\right)\ \mathrm{where}\ \mathrm{a}\ \mathrm{a}\mathrm{nd}\ \mathrm{b}\in \left\{0,1\right\}} $$

*n*_*ab*_ being the occurrence of the following pipeline result combination: result *a* from pipeline A and result *b* from pipeline B, *I* being an indicator function that returns value 1 if the condition into brackets is met, 0 otherwise.

A 2 by 2 contingency table can be fitted to a log-linear model with as much parameters as cells (“saturated model”) [28]:

2 $$ \log \left({n}_{ab}\right)=\mu + a{\lambda}^A+ b{\lambda}^B+ a b\theta $$

On the basis of this equation, ${\widehat{n}}_{ab}$ is the expected occurrence of classification (a,b). Let $\widehat{\mu}$ be the log of the number of chromosomal positions identified as non-variants by both pipelines: $\widehat{\mu}= \log \left({n}_{00}\right)$. Let ${\widehat{\lambda}}^A$ and ${\widehat{\lambda}}^B$ be the logs of the ratios of the number of positions identified as variants by pipelines A and B, respectively, divided by the number of positions identified as non-variants by both pipelines: ${\widehat{\lambda}}^A= \log \left(\frac{n_{10}}{n_{00}}\right)$ and ${\widehat{\lambda}}^B= \log \left(\frac{n_{01}}{n_{00}}\right)$. The estimated odds ratio (OR) for agreement is given by $O\widehat{R}=\frac{n_{11}{n}_{00}}{n_{10}{n}_{01}}= \exp \left(\widehat{\theta}\right)$.

To be able to use proportions instead of numbers of variants and non-variants, an offset was added to most models; it corresponds to the log of the total number of bases (n.. = K on Fig. 1a). This is especially important in the comparisons with Sanger sequencing because the patients did not have the same number of bases sequenced.

#### Pipeline comparison without gold standard

Pipeline comparison was performed considering the pipelines as raters and applying methods developed to analyse inter-rater agreements [29]. The aim was to determine whether two pipelines agree on the number of variants identified (marginal homogeneity), on the identification of variants at the same chromosomal positions (agreement on position), and on the identification of exactly the same variant (with the same alternative proposition in the VCF file) at a specific chromosomal position.

Each patient was considered as a separate study; this led to analyse the results from all patients as a meta-analysis. Thus, 43 independent 2 by 2 tables for agreement (one for each patient) were simultaneously used to analyse the agreement on the presence of variants at the same chromosomal positions. The agreement between two raters (pipelines) was analysed using a two-category classification (variants vs. non-variants). The number of nucleotides sequenced theoretically by the MPS sequencer is n.. = K (Fig. 1a). This led to calculate the number of non-variants n_00_ as the difference between n.. and the total number of variants identified by each pipeline (n_11_ + n_01_ + n_10_). Log-linear models were used to analyse separately marginal and conditional agreements. Comparisons between the nested models using a likelihood ratio test (LRT) led to the choice of the final model.

Let p = 1,…,P be the number of patients. For the meta-analysis, the data were structured in 2 × 2 × P tables. In this case, the saturated model (Eq. 2) becomes:

3 $$ \log \left({n}_{p ab}\right)={\mu}_p+ a{\lambda}_p^A+ b{\lambda}_p^B+ a b{\theta}_p $$

First, a perfect agreement between pipelines implies having the same margins. The general expression of the “homogeneous-margin model” in which *λ*_*p*_^*A*^ and *λ*_*p*_^*B*^ in Eq. 3 are equal is:

4 $$ \log \left({n}_{p ab}\right)={\mu}_p+\left\{ a\left(1- b\right)+ b\left(1- a\right)\right\}{\delta}_p+ ab{\theta}_p $$

where *δ*_*p*_ is the parameter that corresponds to the shared margins.

Second, we defined a model where all patients (or studies) shared a common OR for agreement:

5 $$ \log \left({n}_{p ab}\right)={\mu}_p+ a{\lambda}_p^A+ b{\lambda}_p^B+ a b\theta $$

Third, we defined a model where all patients shared a common intercept:

6 $$ \log \left({n}_{p ab}\right)=\mu + a{\lambda}_p^A+ b{\lambda}_p^B+ a b{\theta}_p $$

The previous three models were compared with the saturated model (Eq. 3) using the LRT. In all tests (2-tailed), the test statistic was compared to a chi-square with the corresponding degrees of freedom (df). A *p* value <5% was considered for statistical significance.

The finally retained model that resulted from the above comparisons was developed into a mixed-effect model with one fixed effect for each parameter and one random effect for the parameters that vary between patients. The mixed-effect model was applied to all 2 × 2× tables to obtain an estimate of the mean of each parameter and an estimate of the variance of each random effect. To obtain easily the number of variants identified by each pipeline (and its confidence interval, CI), we built a re-parameterized mixed model that estimated the parameters of the margins of the 2 × 2 × P tables (See Additional files 1 and 2). The mean marginal probabilities, the mean OR, and the corresponding confidence intervals (CI) were calculated from the estimated parameters and standard errors using a normal approximation. Similarly, biological variability intervals (BVIs) were calculated from the estimated parameters and the random-effect standard deviations using a normal approximation.

**Fig. 2:**
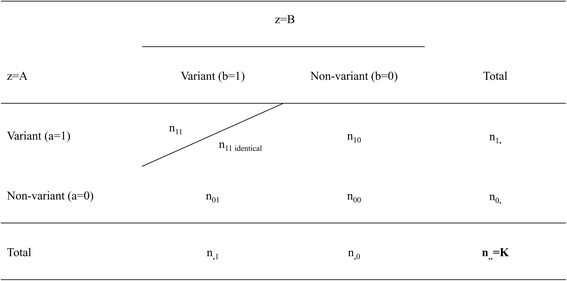
Five-cell contingency table for pipeline agreement on K chromosomal positions and on variant “identity”, without the reference to a gold standard

Knowing that two pipelines have identified a given variant at a given position, we tested this variant “identity”; i.e., whether the variant is really the same (i.e., same reference and alternative proposition in VCF files). A 5-cell contingency table –that identifies the number of identical variants in *n*_11_ cell (Fig. 2) was built and modelled using:

7 $$ \log \left({n}_{p ab}\right)={\mu}_p+ a{\lambda}_p^A+ b{\lambda}_p^B+ a b\left({\theta}_p+ I{\theta}_{p s}\right) $$

where *I* is an indicator taking value 1 when the variants are the same at a given chromosomal position, 0 otherwise and exp(*θ*_*ps*_) the conditional probability associated with the variant “identity”; i.e., knowing that the variants have the same position, this conditional probability is the probability that the variants are identical. To complete the information given by the comparisons between Model 3 (described by Eq. 3) and Models 4 to 6 (described by Eqs. 4 to 6), a log-linear model with a single parameter *θ*_*s*_ for all patients was fitted (Eq. 8 below) and compared with Eq. 7:

8 $$ \log \left({n}_{p ab}\right)={\mu}_p+ a{\lambda}_p^A+ b{\lambda}_p^B+ a b\left({\theta}_p+ I{\theta}_s\right) $$

Finally, the model resulting from the latter comparison was developed into a mixed-effect model and applied to the 2 × 2 × P tables to estimate the mean conditional probability exp(*θ*_*s*_) with its confidence interval and biological variability interval.

#### Pipeline comparison with Sanger sequencing as gold standard

The comparison with the gold standard allows obtaining the sensitivity and specificity of each pipeline. Within this context, sensitivity is the probability of detecting a variant at a given position with a given pipeline knowing that the gold standard has detected a variant at this position (later referred to as “Sanger variant”) whereas specificity is the probability of not detecting a variant at a given position with a given pipeline knowing that the gold standard has not detected a variant at this position (later referred to as “Sanger non-variant”). Thus, comparison of sensitivities and specificities were performed working on Sanger variants and Sanger non-variants, respectively. The contingency table that contains the results of the two pipelines (Fig. 1a) was split up in two contingency tables: the first containing Sanger variants (Fig. 1b) and the second Sanger non-variants (Fig. 1c).

To estimate the sensitivity and specificity of each pipeline, the same analysis described in section “Pipeline comparison without gold standard” was run again: a “homogeneous-margin” model, a model with single parameter for OR of agreement, and a model with single intercept were fitted and compared with a saturated model. The model that resulted from the above comparisons was developed into a mixed-effect model applied to the 2 × 2× P tables. However, to estimate directly the sensitivities and specificities with their corresponding confidence intervals, the latter model was re-parameterized as described above. The confidence intervals were computed using a normal approximation. The BVIs were calculated from the estimated parameters and random-effect standard deviations using a normal approximation. When an estimation of a given parameter was close to one, the normal approximation was not adequate; the confidence intervals were then estimated using a bootstrap percentile method with non-parametric resampling (1000 samples) [30].

Comparisons of the sensitivities and specificities of the two pipelines were carried out by comparing the margins of their 2 × 2 contingency tables. This is equivalent to a classical study of discordant pairs (McNemar test for 2 by 2 tables).

#### Data preparation and model specification

For each patient *p*, the results of the two pipelines (VCF files) were summarized into a response variable that contains the number of variants identified by both pipelines A and B (n_p11_, common variants), the number of variants identified by pipeline A only (n_p10_), the number of variants identified by pipeline B only (n_p01_), and the number of non-variants (n_p00_) (Fig. 1a). The number of non-variants was the difference between the number of bases sequenced and the total number of variants identified: n_p00_ = n_p.._ - (n_p11_ + n_p10_ + n_p01_). To build the log-linear models, we created several dummy variables that correspond to the model parameters. A first dummy variable that takes value 1 when the response variable corresponds to common variants to both pipelines (0 otherwise) was used to estimate parameters θ or θ_p_. A second dummy variable that takes value 1 when the response variable corresponds to variants found by pipeline A (0 otherwise) was used to estimate parameter *λ*_*p*_^*A*^. A third dummy variable that takes value 1 when the response variable corresponds to variants found by pipeline B (0 otherwise) was used to estimate parameter *λ*_*p*_^*B*^. To build the homogeneous-margin model, a fourth dummy variable that takes value 1 when the response variable corresponds to variants identified by pipeline A or B (0 otherwise) was used to estimate parameter δ_p_.

For the 5-cell contingency tables, when we wanted to estimate the number of “identity” variants, we added to the response variable the number of variants common to the two pipelines (i.e., same reference and alternative proposition in VCF files). To estimate parameter θ_s_, we created a dummy variable that takes value 1 when the response variable corresponds to “identity” variants (0 otherwise).

The same data structuring was used to analyse the results of the pipelines knowing the gold standard results but, here, only the positions sequenced by Sanger method and identified as variants were considered to estimate the sensitivity and, similarly, only the positions sequenced by Sanger and identified as non-variants were considered to estimate the specificity.

All analyses were carried out with R software. Log-linear models were fitted with *glm* function using a Poisson distribution; these models included the adequate dummy variables. The mixed models that correspond to the finally retained models were fitted with *glmer* function of *lme4* package with Poisson distribution. The LRT was applied with *lrtest* function of *lmtest* package. The same statistical analyses were carried out first on all variants identified by each pipeline then only on SNVs.

Further details and code examples are available as Additional files 1 and 2.

## Results

### Data description

The MPS sequencing covered 41 genes over 390339 base-pairs per patient. For each patient, the MPS sequencing provided a list of variants obtained by BWA-GATK and another list obtained by TMAP-NextGENe®. Each list included nearly 2000 variants of which 300 SNVs (Table 1).

**Table 1:** Number of variants identified by the pipelines and by the gold standard in N patients

Variants and pipelines	N	Mean ± SD	Min.	Q1	Median	Q3	Max.
All types of variants^a^							
Regions sequenced by MPS only^b^							
BWA-GATK	43	1871 ± 225.08	1198	1696	1867	2056	2360
TMAP-NextGENe	43	2280 ± 339.72	1214	2014	2256	2504	3094
Regions sequenced by MPS + gold standard^b^							
Sanger	30	2.67 ± 2.88	0	1	1	3.75	10
BWA-GATK	30	27.40 ± 20.54	3	10.50	25	36	92
TMAP-NextGENe	30	22.77 ± 18.71	3	9.25	17	28	75
SNVs only							
Regions sequenced by MPS only^a^							
BWA-GATK	43	267 ± 22.04	204	251.50	267	280	318
TMAP-NextGENe	43	315.10 ± 28.32	215	302.50	317	334	384
Regions sequenced by MPS + gold standard^b^							
Sanger	30	2.30 ± 2.79	0	0	1	3	9
BWA-GATK	30	2.77 ± 2.81	0	1	2	4	10
TMAP-NextGENe	30	2.77 ± 2.81	0	1	2	4	10

^a^Single Nucleotide Variants (SNVs), insertions, and deletions

^b^390,339 base-pairs per patient

^c^1,085 to 16,570 base-pairs per patient

In our comparisons with Sanger sequencing, we considered only the genes sequenced by both Sanger and MPS; i.e., 1 to 3 genes (1085 to 16570 base-pairs) per patient. In this case, the number of variants decreased to an average of 25, of which an average of three SNVs per patient. Depending on the number of sequenced genes, the Sanger sequencing list included 0 to 9 variants.

### Analysis of all types of variants (SNVs, deletions, and insertions)

#### BWA-GATK vs. TMAP-NextGENe® comparison without gold standard

We investigated first whether BWA-GATK and TMAP-NextGENe® could identify variants at the same chromosomal positions. Comparing the saturated vs. the homogeneous-margin model, the pipelines had distinct margins within each table (LRT with 43 df, *p* value <0.001). Comparing the saturated vs. the common-OR model, the ORs for agreement were different between patients (LRT with 42 df, *p* value <0.001). Using the re-parameterized model implied using the same intercept for all patients because the same number of bases were sequenced; this led to a common-intercept model. When, the mixed-effect model that corresponds to the latter model was fitted, BWA-GATK identified, on average, 1857.14 variants (95% CI: [1789.27; 1927.58] and 95% BVI: [1461.16; 2360.43]) whereas TMAP-NextGENe® identified 2253.49 variants (95% CI: [2149.96; 2361.99] and 95% BVI: [1660.16; 3058.86]). The mean OR for agreement was estimated at 497.08 (95% CI: [464.55; 531.89]), with between-patient 95% BVI: [323.36; 764.13] (see Table 2).

**Table 2:** Results of pipeline comparisons

	Parameter estimation		
Variants, pipelines, and parameters	Value	[95% CI]	[95% BVI]
All types of variants			
Regions sequenced by MPS only^a^			
Number of variants for BWA-GATK	1857.14	[1789.27; 1927.58]	[1461.16; 2360.43]
Number of variants for TMAP-NextGENe	2253.49	[2149.96; 2361.99]	[1660.16; 3058.86]
OR for agreement	497.08	[464.55; 531.89]	[323.36; 764.13]
Conditional probability of identity	0.24	[0.23; 0.25]	[0.20; 0.28]
Regions sequenced by MPS and gold standard^b^			
Sensitivity of BWA-GATK (%)	63.47	[46.01; 87.55]	[44.91; 89.69]
Sensitivity of TMAP-NextGENe (%)	63.42	[45.95; 87.53]	[44.68; 90.02]
FP rate for 10,000 Sanger NV for BWA-GATK	43.03	[41.22; 45.20]	NA
FP rate for 10,000 Sanger NV for TMAP-NextGENe	35.25	[33.59; 36.80]	NA
Only SNVs			
Regions sequenced by MPS only^a^			
Number of SNVs for BWA-GATK	266.41	[259.17; 273.84]	[232.84; 304.81]
Number of SNVs for TMAP-NextGENe	314.24	[305.52; 323.21]	[271.11; 364.24]
OR for agreement	23289.85	[20226.42; 26817.25]	[10099.35; 53708.09]
Conditional probability of identity	0.9986	[0.9984;0.9989]	NA
Regions sequenced by MPS and gold standard^b^			
Sensitivity of BWA-GATK (%)	76.81	[63.50; 92.92]	NA
Sensitivity of TMAP-NextGENe (%)	76.81	[63.50; 92.92]	NA
FP rate for 10,000 Sanger NS for BWA-GATK	2.01	[1.82; 2.24]	NA
FP rate for 10,000 Sanger NS for TMAP-NextGENe	2.01	[1.82; 2.24]	NA

*CI* Confidence Interval, *BVI* Biological Variability Interval, *OR* Odds Ratio, *FP* False Positive, *NV* Non-variants, *NS* Non-SNVs

^a^390,339 base-pairs per patient

^b^1,085 to 16,570 base-pairs per patient

We then investigated whether BWA-GATK and TMAP-NextGENe® could identify exactly the same variants at the same positions. Comparing the saturated identity-model (Eq. 7) vs. the common-identity model (Eq. 8), the parameters of variant “identity” were different between patients (LRT with 42 df, *p* value <0.001); this led to retain the model with common intercept but different parameters of variant “identity” between patients. Providing that the two pipelines identified one variant at a given chromosomal position, the estimated probability that this variant would be exactly the same was 0.24 (95% CI: [0.23; 0.25] and its 95% BVI: [0.20; 0.28]).

#### BWA-GATK vs. TMAP-NextGENe® comparison with gold standard

Regarding the analysis of Sanger non-variants, the margins were significantly different (LRT with 30 df, *p* value <0.001); consequently, the specificities of the two pipelines were statistically significantly different despite very close values. The ORs for agreement were significantly different between patients (LRT with 29 df, *p* value = 0.044) whereas the intercepts were not significantly different (LRT with 29 df, *p* value = 1); this led to retain the model with a single intercept. When, the common-intercept mixed-effect model was used, the BWA-GATK specificity was 99.57% (95% CI: [99.55%; 99.59%]) and the TMAP-NextGENe® specificity 99.65% (95% CI: [99.63%; 99.66%]). A very small between-patient variability was found with each pipeline; i.e., no biological variability could be estimated. The specificities being very high due to the tremendous number of non-variants, the corresponding FP rates was deemed to be a more interesting parameter than specificity. For 10,000 positions identified as non-variant with Sanger sequencing, the estimated number of FPs was 43.03 (95% CI: [41.22; 45.20]) with BWA-GATK and 35.25 (95% CI: [33.59; 36.80]) with TMAP-NextGENe® (see Table 2).

When Sanger variants were considered, their number being low, comparison tests using nested models were not pertinent because of their low power. We chose then to use the same mixed model we used with Sanger non-variants. The sensitivities of the two pipelines were very close: 63.47% (95% CI: [46.01%; 87.55%] and 95% BVI [44.91%; 89.69%]) for BWA-GATK vs. 63.42% (95% CI: [45.95%; 87.53%] and 95% BVI [44.68%; 90.02%]) for TMAP-NextGENe® (see Table 2).

### Analysis of SNVs only

#### BWA-GATK vs. TMAP-NextGENe®: comparison without gold standard

We investigated first whether BWA-GATK and TMAP-NextGENe® could identify SNVs at the same chromosomal positions. Model comparisons showed that the margins were different between pipelines (LRT with 43 df, *p* value <0.001), the ORs for agreement were significantly different between patients (LRT with 42 df, *p* value <0.001), and the intercepts were not significantly different (LRT with 42 df, p value = 1); this led to retain the model with a single intercept. When, the corresponding mixed-effect model was fitted, BWA-GATK and TMAP-NextGENe® identified, on average, 266.41 and 314.24 SNVs, respectively (95% CIs: [259.17; 273.84] and [305.52; 323.21] and 95% BVIs: [232.84; 304.81] and [271.11; 364.24], respectively). The estimated mean OR for agreement was 23289.85 (95% CI: [20226.42; 26817.25] and 95% BVI: [10099.35; 53708.09]) (see Table 2).

We then investigated whether BWA-GATK and TMAP-NextGENe® could identify exactly the same SNVs at the same positions. We found that the parameter for variant “identity” was not significantly different between patients (LRT with 42 df, *p* value = 1), which led to retain a model with a common intercept and a common parameter for variant “identity”. Providing that the two pipelines identified one SNV at a given chromosomal position, the estimated probability that this SNV would be exactly the same was 0.9986 (95% CI: [0.9984; 0.9989]) (see Table 2).

#### BWA-GATK vs. TMAP-NextGENe®: comparison with gold standard

Regarding the analysis of non-SNVs identified by Sanger, i) the margins were not significantly different (LRT with 30 df, p value = 0.07); ii) the ORs for agreement and the intercepts were common between patients (LRT with 29 df, *p* value = 0.894 and LRT with 29 df, *p* value = 1, respectively). This led to retain the “homogeneous-margin” model with common intercept and OR. BWA-GATK and TMAP-NextGENe® had the same specificity: 99.98% (95% CI: [99.9776%; 99.9819%]). Over 10,000 non-variant positions identified with Sanger sequencing, the estimated number of FPs was 2.01 (95% CI: [1.82; 2.24]) for BWA-GATK and TMAP-NextGENe® (see Table 2).

When we analysed the SNVs identified by Sanger sequencing, the same above-mentioned reasons (very few SNVs and low power) led us to use the same mixed model as with Sanger non-variants. The estimated sensitivity was then 76.81% (95% CI: [63.50%; 92.92%]) for BWA-GATK and TMAP-NextGENe® (see Table 2).

## Discussion

Currently, a large number of pipelines are being developed to analyze MPS data. Choosing a pipeline is often very difficult; it is thus important to develop statistical methods to compare the results given by various pipelines. In addition, for diagnostic purposes, the sensitivity and specificity of the diagnostic test should be assessed. We thus developed a statistical method to compare MPS pipelines and assess the quality of their results.

Taking advantage of available data on epileptic patients, we designed a strategy to compare two MPS data analysis pipelines. We considered the genomic position as the statistical unit, each patient as a separate study, and the analysis of all patients as a meta-analysis. The method was applied first to all variants then to SNVs only. Furthermore, we compared two pipelines without considering a gold standard then compared the same two pipelines versus Sanger sequencing as a gold standard. Finally, to put the precision of the estimates within the context of patient heterogeneity, we gave a biological variability interval between patients.

Overall, the results demonstrated that the performance of BWA-GATK was very close to that of TMAP-NextGENe® but that the performance of each changed according to the type of variants considered (indels and/or SNVs). When all types of variants were considered, the estimate of the OR for agreement was very high, which means a strong agreement between the two pipelines. The sensitivities were estimated around 63% and the specificities around 99%. The estimated specificities being close to 1, the corresponding FP rates seemed more useful for the comparison: BWA-GATK identified a slightly higher number of FPs than TMAP-NextGENe® (43 vs. 35 for 10,000 non-variant positions with Sanger sequencing). The confidence intervals of the estimated sensitivities were similar between the two pipelines but both very wide because of the small number of patients and the small number of variants. Also, both biological variability intervals were very wide, which means that the performances of the two pipelines are very dependent on the biological variability; i.e., on the patient mix.

When only SNVs were analysed, the number of SNVs per patient being small, the performances of the two pipelines could not be statistically different. In addition, with the two pipelines, the number of FPs decreased strongly, the sensitivities increased and the OR for agreement increased. The latter result (a stronger agreement with SNVs only than with all variants combined) was expected because it is well known that pipelines are better at detecting SNVs than other variants. This can be partly explained by the facts that: (i) MPS technologies, particularly Ion Torrent PGM™, have difficulties in sequencing DNA regions containing homopolymers, which leads to the creation of “false” indels; and (ii) alignment on Hg19 is more complex in regions with homopolymers than in other regions, which leads the two pipelines to find more FPs in these regions than in others [19]. The number of FPs, though smaller with SNVs only than with all variants combined, remained nevertheless high with regard to the number of positions in the whole genome.

When not only the positions but also the variant “identities” were considered, the results confirmed the difficulties of MPS technologies in identifying indels. Indeed, most SNVs found by the two pipelines at the same positions were identical. On the contrary, investigating all types of variants, most variant “identities” found by the two pipelines at the same positions were different; e.g., there were either SNVs instead of insertions or insertions of three bases instead of four.

Overall, TMAP-NextGENe® gave slightly better results than BWA-GATK because, with the same sensitivity, the former generated less FPs. This may be explained by the TMAP alignment which was adapted by Life technology to correct the main weaknesses of the Ion Torrent technology.

In this paper, we studied the intrinsic performance of each pipeline; i.e., its sensitivity and specificity. By definition, these indicators do not depend on the prevalence of the variants. When a pipeline is designed to analyse NGS data in a diagnostic context, its positive and negative predictive values (PPV and NPV) should also be determined. Within this context, the PPV is the probability that a detected variant is really a variant and the NPV the probability that a non-variant is really a non-variant. The positive and negative predictive values depend on both the intrinsic performance and the prevalence of the variants; thus on the disease under study. For example, with the two studied pipelines, considering a prevalence of 5 variants for 10,000 positions, the PPV of BWA-GATK was 88.58%, the PPV of TMAP-NextGENe® was 90.51%, and the NPV was 98.10% for both pipelines.

The statistical method presented here can be used to compare any two pipelines. The results of the LRT should not be the only criteria to consider for choosing the mixed model because these results are very dependent on the sample size. When the number of variants identified by the gold standard method is small, the LRT is not powerful enough to reveal a difference in sensitivity between two pipelines. In this case, it seems more relevant to apply either the same model as the one chosen for specificity or another model recommended by the literature.

With the increasing use of MPS in diagnostic laboratories, the development of statistical methods to compare pipelines is essential. Several tools already exist to compare pipeline results: VCFtools or the more recent GCAT Benchmarking tool [31] and RTG Tools [32], for example. Briefly, RTG tools take into account the “complex call representation” found by variant calling. GCAT Benchmarking tool offers a pleasant interface to compare alignment results or variant callers and uses its proper gold standard to calculate sensitivities and specificities and produce ROC-like curves. These tools are very useful and important to begin any analysis and may be used to complete our method. Generally, the validation of new pipelines or new versions of already existing pipelines requires extensive comparisons with robust statistical methods. The simple sensitivity and specificity calculations often used in pipeline validation describe the sample under study but cannot be valid in future subjects, especially when small samples are used for pipeline validation. These calculations are sensitive to outliers and do not allow estimating the variability between patients, which may be very high. The statistical method proposed in this paper allows estimating non-biased performance indicators (sensitivity and specificity) and estimate their agreement (OR). In addition, this method allows a valid transposition of pipeline experimental results to the general population while taking into account the variability between patients and/or sequenced genes. Moreover, a statistical model should allow introducing covariates such as the sequencing depth or the genome guanine-cytosine content. Here, for simplicity, we did not use such covariates but, in further works with diagnostic purposes, introducing covariates to characterize variant positions seems interesting, if not essential.

Up to now, Sanger sequencing has been the reference method in medical research. This is why we considered it here as gold standard though we are aware that its results do not always reflect the biological truth. Statistical methods have been developed to estimate sensitivity and specificity in case of imperfect gold standard [33]. These methods may be extended to the field of pipeline assessment. We mention here that the statistical method we present does not depend on the choice of the gold standard: the same analysis may be performed with any other gold standard than Sanger sequencing. Another limit with Sanger sequencing is the small number of genes sequenced, thus the small number of identifiable variants; this leads to a low power in comparing pipeline sensitivities.

In the present paper, we carried out an overall comparison of two pipelines using the results of sequencing a panel of genes. However, the method may be used for the comparison of particular pipeline steps or options and for analyses of exomes or whole genomes. In the future, this method will be extended to comparisons between more than two pipelines.

Two other important steps in MPS data analysis are variant calling and filtering. In this study, we discarded only the variants whose depth of coverage was <10×. We have chosen not to annotate and filter the variants identified by the two pipelines before comparing their raw VCF files. The addition of an annotation and filtering step would have certainly reduced the number of FPs but with the risk of eliminating true variants and, thus, decreasing the estimated sensitivities. The exact impact of the filtering step may be the object of future studies.

## Conclusion

In conclusion, the statistical method we propose in this paper showed that the commercial pipeline (TMAP-NextGENe®) gave slightly better results than the academic pipeline (BWA-GATK) because, with the same sensitivity, the former generated less FPs. The method allows choosing the most appropriate pipeline for a given analysis and is generalizable to all types of pipelines and MPS data (panel, exome, whole genome) that are becoming increasingly used for diagnosis, prognosis, and therapeutics in the evolving personalized medicine.

## Additional files

Additional file 1: R code example. This R code allows reproducing the findings presented in the article regarding comparison results between two pipelines (BWA-GATK and TMAP-NextGen) without taking into account a Gold Standard (here, Sanger sequencing). When Gold Standard results are available, some data preparation steps should be added before modelling. All the details about these steps are given in the R file. (R 16 KB) (R 15 kb)
Additional file 2: Pipeline results. This Rdata, which is loaded in R code file, contains pipeline results for BWA-GATK (object *BWAPat*) and TMAP-NextGen (object *NGPat*) as well as region sequenced (object *BedNGS*). (RData 8 MB) (RDATA 8590 kb)

## Abbreviations

BAM: Binary alignment map
BVI: Biological variability interval
BWA: Burrows-wheeler aligner
CI: Confidence interval
DF: Degrees of freedom
DNA: Deoxyribonucleic acid
FP: False positive
GATK: Genome alignment toolkit
GCAT: Genome comparison and alignment tool
LRT: Likelihood ratio test
MPS: Massive parallel sequencing
NGS: Next generation sequencing
NPV: Negative predictive value
OR: Odds ratio
PCR: Polymerase chain reaction
PGM: Personal genome machine
PPV: Positive predictive value
ROC: Receiver operating characteristic
RTG: Real time genomics
SNV: Single nucleotide variant
TMAP: Torrent mapping alignment program
VCF: Variant call format

## References

[1] Sanger F, Nicklen S, Coulson AR (1977). DNA sequencing with chain-terminating inhibitors. Proc Natl Acad Sci U S A.

[2] Metzker ML (2010). Sequencing technologies - the next generation. Nat Rev Genet.

[3] Liu L, Li Y, Li S (2012). Comparison of next-generation sequencing systems. J Biomed Biotechnol.

[4] Chrystoja CC, Diamandis EP (2014). Whole genome sequencing as a diagnostic test: challenges and opportunities. Clin Chem.

[5] Harismendy O, Ng PC, Strausberg RL (2009). Evaluation of next generation sequencing platforms for population targeted sequencing studies. Genome Biol.

[6] Quail M, Smith ME, Coupland P (2012). A tale of three next generation sequencing platforms: comparison of Ion torrent, pacific biosciences and illuminaMiSeq sequencers. BMC Genomics.

[7] Archer J, Weber J, Henry K, et al. Use of Four Next-Generation Sequencing Platforms to Determine HIV-1 Coreceptor Tropism. Plos One. 2012;s7(11). 10.1371/journal.pone.0049602 PMC3498215 23166726

[8] Oliver GR, Hart SN, Klee EW (2015). Bioinformatics for clinical next generation sequencing. Clin Chem.

[9] Langmead B, Trapnell C, Pop M, et al. Ultrafast and memory-efficient alignment of short DNA sequences to the human genome. Genome Biol. 2009;10:R25. 10.1186/gb-2009-10-3-r25 PMC2690996 19261174

[10] Li H, Ruan J, Durbin R (2008). Mapping short DNA sequencing reads and calling variants using mapping quality scores. Genome Res.

[11] Li H, Durbin R (2009). Fast and accurate short read alignment with Burrows-Wheeler transform. Bioinformatics.

[12] Li R, Li Y, Kristiansen K (2008). SOAP: Short oligonucleotide alignment program. Bioinformatics.

[13] McKenna A, Hanna M, Banks E (2010). The genome analysis toolkit: a MapReduce framework for analyzing next-generation DNA sequencing data. Genome Res.

[14] Li H, Handsaker B, Wysoker A (2009). The sequence alignment/Map format and SAMtools. Bioinformatics.

[15] Garrison E, Marth G. Haplotype-based variant detection from short-read sequencing. arXiv 1207.3907v2 [q-bio.GN] 12 Jul 2012.

[16] Gomez J, Reguero JR, Moris C (2014). Mutation analysis of the main hypertrophic cardiomyopathy genes using multiplex amplification and semiconductor next-generation sequencing. Circ J.

[17] Sikkema-Raddatz B, Johansson LF, De Boer EN (2013). Targeted next-generation sequencing can replace Sanger sequencing in clinical diagnostics. Hum Mutat.

[18] Castera L, Krieger S, Rousselin A (2014). Next-generation sequencing for the diagnosis of hereditary breast and ovarian cancer using genomic capture targeting multiple candidate genes. Eur J Hum Genet.

[19] Tarabeux J, Zeitouni B, Moncoutier V (2013). Streamlined ion torrent PGM-based diagnostics: BRCA1 and BRCA2 genes as a model. Eur J Hum Genet.

[20] Millat G, Chanavat V, Rousson R (2014). Evaluation of a New high-throughput next-generation sequencing method based on a custom AmpliSeqTM library and Ion torrent PGM™ sequencing for the rapid detection of genetic variations in long QT syndrome. Mol DiagnTher.

[21] Singh RR, Patel KP, Routbort MJ (2013). Validation of a next-generation sequencing screen for mutational hotspots in 46 cancer-related genes. J Mol Diagn.

[22] Daber R, Sukhadia S, Morrissette JJD (2014). Understanding the limitations of next generation sequencing informatics, an approach to clinical pipeline validation using artificial data sets. Cancer Genet.

[23] Nevado B, Perez-Enciso M (2015). Pipeliner: software to evaluate the performance of bioinformatics pipelines for Next Generation re-Sequencing. Mol Ecol Resour.

[24] Van der Auwera GA, Carneiro M, Hartl C (2013). From FastQ data to high-confidence variant calls: the genome analysis toolkit best practices pipeline. Curr Protoc Bioinformatics.

[25] Li H, Durbin R (2010). Fast and accurate long-read alignment with Burrows-Wheeler transform. Bioinformatics.

[26] Ning Z, Cox AJ, Mullikin JC (2001). SSAHA: a fast search method for large DNA databases. Genome Res.

[27] Li H (2012). Exploring single-sample SNP and INDEL calling with whole-genome de novo assembly. Bioinformatics.

[28] Agresti A. Categorical Data Analysis, 3rd edition. Hoboken, NJ: Wiley; 2013.

[29] Becker MP, Agresti A (1992). Log-linear modelling of pairwise interobserver agreement on a categorical scale. Stat Med.

[30] Carpenter B, Bithell J (2000). Bootstrap confidence intervals: when, which, what? a practical guide for medical statisticians. Stat Med.

[31] Highnam G, Wang JJ, Kusler D (2015). An analytical framework for optimizing variant discovery from personal genomes. Nat Commun.

[32] Cleary JG, Braithwaite R, Gaastra K, et al. Comparing Variant Call Files for Performance Benchmarking of Next-Generation Sequencing Variant Calling Pipelines. bioRxiv 023754; doi: https://doi.org/10.1101/023754

[33] Zhou XH, Obuchowski NA, McClish DK (2002). Statistical methods in diagnostic medicine.

